# Altered exocytosis of inhibitory synaptic vesicles at single presynaptic terminals of cultured striatal neurons in a knock-in mouse model of Huntington’s disease

**DOI:** 10.3389/fnmol.2023.1175522

**Published:** 2023-08-17

**Authors:** Chen Xu, Sidong Chen, Xingxiang Chen, Ka Hei Ho, Chungwon Park, Hanna Yoo, Suk-Ho Lee, Hyokeun Park

**Affiliations:** ^1^Division of Life Science, The Hong Kong University of Science and Technology, Kowloon, Hong Kong SAR, China; ^2^Hong Kong Center for Construction Robotics (InnoHK-HKCRC), Hong Kong Science Park, Sha Tin, Hong Kong SAR, China; ^3^Department of Physiology, Seoul National University College of Medicine, Seoul, Republic of Korea; ^4^Department of Physics, The Hong Kong University of Science and Technology, Kowloon, Hong Kong SAR, China; ^5^State Key Laboratory of Molecular Neuroscience, The Hong Kong University of Science and Technology, Kowloon, Hong Kong SAR, China

**Keywords:** Huntington’s disease, synaptic vesicle, exocytosis, inhibitory synapses, real-time imaging, presynaptic terminal

## Abstract

Huntington’s disease (HD) is a progressive dominantly inherited neurodegenerative disease caused by the expansion of a cytosine-adenine-guanine (CAG) trinucleotide repeat in the *huntingtin* gene, which encodes the mutant huntingtin protein containing an expanded polyglutamine tract. One of neuropathologic hallmarks of HD is selective degeneration in the striatum. Mechanisms underlying selective neurodegeneration in the striatum of HD remain elusive. Neurodegeneration is suggested to be preceded by abnormal synaptic transmission at the early stage of HD. However, how mutant huntingtin protein affects synaptic vesicle exocytosis at single presynaptic terminals of HD striatal neurons is poorly understood. Here, we measured synaptic vesicle exocytosis at single presynaptic terminals of cultured striatal neurons (mainly inhibitory neurons) in a knock-in mouse model of HD (zQ175) during electrical field stimulation using real-time imaging of FM 1-43 (a lipophilic dye). We found a significant decrease in bouton density and exocytosis of synaptic vesicles at single presynaptic terminals in cultured striatal neurons. Real-time imaging of VGAT-CypHer5E (a pH sensitive dye conjugated to an antibody against vesicular GABA transporter (VGAT)) for inhibitory synaptic vesicles revealed a reduction in bouton density and exocytosis of inhibitory synaptic vesicles at single presynaptic terminals of HD striatal neurons. Thus, our results suggest that the mutant huntingtin protein decreases bouton density and exocytosis of inhibitory synaptic vesicles at single presynaptic terminals of striatal neurons, causing impaired inhibitory synaptic transmission, eventually leading to the neurodegeneration in the striatum of HD.

## Introduction

Huntington disease (HD) is a progressive dominantly inherited neurodegenerative disease caused by abnormally expanded cytosine-adenine-guanine (CAG) trinucleotide repeats in the first exon in the *huntingtin* (*HTT*) gene (The Huntington’s Disease Collaborative Research Group, 1993), which encodes an anomalously expanded polyglutamine (polyQ) tract in the huntingtin protein. The symptoms of HD include abnormal involuntary movements, psychiatric disturbance, and cognitive impairment (Roos, 2010; Dayalu and Albin, 2015; McColgan and Tabrizi, 2018). Although the huntingtin protein is distributed throughout the body, inhibitory medium spiny neurons (MSNs) in the striatum are the most vulnerable neurons in HD (Vonsattel and DiFiglia, 1998).

Selective neurodegeneration of striatal neurons in HD was suggested to be associated with abnormal synaptic transmission (Klapstein et al., 2001; Cepeda et al., 2003; Li et al., 2003; Smith et al., 2005; Raymond et al., 2011; Tyebji and Hannan, 2017; Chen et al., 2018; Virlogeux et al., 2018; Barron et al., 2021; Chen S. et al., 2021; Barry et al., 2022; Cepeda and Levine, 2022), reduced availability of brain-derived neurotrophic factor (BDNF) to the striatum (Zuccato and Cattaneo, 2009, 2014; Park, 2018; Yu et al., 2018), impaired mitochondrial function (Carmo et al., 2018; Franco-Iborra et al., 2018; Sawant et al., 2021), and abnormal Ca^2+^ regulation (Bezprozvanny, 2009; Miller and Bezprozvanny, 2010; Raymond, 2016; Chen et al., 2018). Much research has focused on abnormal synaptic transmission in corticostriatal synapses and excitotoxicity in HD (Smith et al., 2005; Raymond, 2016; Smith-Dijak et al., 2019; Cepeda and Levine, 2022). Increased synaptic vesicle release was observed in cortical neurons in young HD mice (Joshi et al., 2009; Chen et al., 2018). Moreover, increased activity of extrasynaptic NMDA receptors was reported in acute slices obtained from the striatum of HD mice (Milnerwood et al., 2010) and was suggested to cause neurodegeneration in the striatum of HD (Milnerwood and Raymond, 2010; Botelho et al., 2014; Ribeiro et al., 2017). In addition to abnormal synaptic transmission in corticostriatal synapses in HD, recent findings suggested that abnormal synaptic transmission in GABAergic synapses may underlie HD pathogenesis (Garret et al., 2018; Hsu et al., 2018). Electrophysiology measurement showed altered striatal synaptic transmission in HD mice (Cummings et al., 2010). Furthermore, the expression level of GABA_A_R subunits was altered in HD mouse models (Du et al., 2017; Paraskevopoulou et al., 2021). However, how the mutant huntingtin protein affects exocytosis of inhibitory synaptic vesicles at single presynaptic terminals of HD striatal neurons is poorly understood.

In this study, we used real-time imaging of FM 1-43-loaded synaptic vesicles to measure synaptic vesicle exocytosis at single presynaptic terminals of cultured striatal neurons during electrical field stimulation. Bouton density and synaptic vesicle exocytosis were decreased at single presynaptic terminals in cultured striatal neurons (mainly inhibitory neurons) of a knock-in mouse model of HD (zQ175). Furthermore, real-time imaging of inhibitory synaptic vesicles containing VGAT-CypHer5E showed a decrease in bouton density and exocytosis of inhibitory synaptic vesicles at single presynaptic terminals in HD cultured striatal neurons. Thus, our results suggest that the mutant huntingtin protein decreases inhibitory bouton density and exocytosis of inhibitory synaptic vesicles at presynaptic terminals and alters synaptic transmission in the striatum at the early stage of HD, leading to selective neurodegeneration in the striatum of HD.

## Materials and methods

### Mice

The zQ175 (a HD knock-in mouse model) mice were purchased from the Jackson Laboratories and were kept in the Animal and Plant Care Facility at the Hong Kong University of Science and Technology. Heterozygous zQ175 mice were utilized for breeding. All experimental procedures for mice were approved by the Department of Health, Government of Hong Kong and were performed following the approved protocols.

### Culturing striatal neurons

Striatal tissue from postnatal day 0 (P0) heterozygous pups and wild-type (WT) littermates was used to culture HD and WT striatal neurons. Culturing striatal neurons was performed similarly as culturing cortical neurons (Alsina et al., 2017; Chen et al., 2018; Chen S. et al., 2021). Dissected striatal tissue was digested with papain (LS003127, Worthington Biochemical Corp., USA) and DNAse (D5025, Sigma-Aldrich, USA). Around 40000 striatal neurons were plated on each 12-mm glass coverslip coated with poly-D-lysine (P7405, Sigma-Aldrich) in 24-well plates (Park et al., 2012; Chen et al., 2018). At 3 days *in vitro* (DIV3), cultured striatal neurons were treated with 20 μM 5-Fluoro-2′-deoxyuridine (FUDR, Sigma) to prevent the proliferation of glia cells. Cultured striatal neurons were grown at 37°C in a humidified incubator containing 5% CO_2_ for at least 14 days. All experiments were performed between DIV14 and DIV16.

### Immunofluorescence

Fixation of cultured striatal neurons was performed using ice-cold 100% methanol for 10 min. After washing with PBS three times and blocking with 5% goat serum in staining buffer (0.2% BSA, 0.8 M NaCl, 0.5% Triton X-100, 30 mM phosphate buffer, pH7.4), 50 μL of primary antibody mixtures containing polyclonal anti-MAP2 (1:1000, Ab5392 (Abcam)), monoclonal anti-DARPP32 (1:500, Ab40801 (Abcam)) and monoclonal anti-GAD67 (1:500, MAB5406 (Merck)) or monoclonal anti-VGLUT1 (1:500, MAB5502 (Merck)) were added to coverslips, and then striatal neurons were incubated at 4°C overnight. Then, following three times washing with PBS for 10 minutes, 1:1,000 diluted secondary antibodies containing goat anti-chicken-Alexa 488 (A11039 (Invitrogen)), goat anti-rabbit Alexa 568 (A11011 (Invitrogen)) and donkey anti-mouse Alexa 647 (A31571 (Invitrogen)) were added to the coverslips and incubate for 1 hour in room temperature in the following day. After washing three times, 250 nM DAPI (D1306 (Invitrogen)) was added and incubated for another 10 minutes at room temperature. After three times washing with PBS, coverslips were mounted on to Glass slides with HydroMount medium. Confocal images were acquired using a SP8 confocal microscope (Leica) with a 40X oil objective. The percentage of GAD67-positive neurons was calculated as the ratio of the number of GAD67-positive cells to the number of MAP2-positive cells. The percentage of VGLUT1-positive neurons was calculated as the ratio of the number of VGLUT1-positive cells to the number of MAP2-positive cells. The percentage of DARPP-32-positive neurons was calculated as the ratio of the number of DARPP-32-positive cells to the number of GAD67-positive cells. The numbers of MAP2-positive, GAD67-positive, VGLUT1-positive, and DARPP-32-positive cells were measured in a blinded manner.

### Imaging of FM 1-43-loaded synaptic vesicles

Loading FM 1-43 into synaptic vesicles in cultured striatal neurons was performed by applying 1,200 external field stimuli at 10 Hz for 120 s in the presence of 16 μM FM 1-43 (T35356 (Thermo Fisher Scientific, USA)) in a sample chamber at 37°C with a platinum electrode wired to a stimulator (SD9 Grass Stimulator (Grass Technologies, USA)) as previously described (Park et al., 2012; Chen et al., 2018; Chen S. et al., 2021). After loading, the sample chamber was perfused for 10 min for washing with artificial cerebrospinal fluid (ACSF) solution containing (in mM): 120 NaCl, 4 KCl, 2 CaCl_2_, 2 MgCl_2_, 10 D-Glucose, and 10 HEPES (300-310 mOsm, pH 7.2-7.4 with NaOH). Exocytosis experiments of FM 1-43-loaded synaptic vesicles were performed as described previously (Chen et al., 2018). Images were obtained for 200 s at 1 Hz with an exposure time of 0.1 s with an EMCCD camera (iXon Ultra (Andor camera)). An experimental setup consisted of several pieces of equipment as described previously (Alsina et al., 2017; Chen et al., 2018). The shutter, stimulator, and camera were synchronized with a trigger from the camera through a Digidata 1550 (Molecular Devices, USA). Clampex (Molecular Devices) was used for generating electrical stimulation protocols. An IX-73 microscope (Olympus) with a 100X oil-immersion objective (UPlanSApo (Olympus)) was used. A 532-nm laser (OBIS 532 (Coherent Inc.)) with a dichroic mirror (ZT532rdc, Chroma) and emission filter (ET595/50m) was used to image FM 1-43-loaded synaptic vesicles. Normalized fluorescence of FM 1-43-loaded synaptic vesicles was calculated as the average fluorescence intensity in a region of interest (ROI) relative to the average fluorescence intensity in the same ROI during the first 20 s before electrical stimulation. The fluorescence intensity was measured with MetaMorph (Molecular Devices). Customer-made MATLAB (MathWorks Inc.) program was used to calculate the normalized fluorescence. Average normalized fluorescence was computed by averaging all normalized fluorescence from individual analyzed presynaptic terminals. Fluorescence loss of FM 1-43-loaded synaptic vesicles at single presynaptic terminals during stimulation was computed by subtracting the average normalized fluorescence in the final 60 s after stimulation from the average normalized fluorescence in first 20 s and was expressed as percentage. The destaining time constant of FM 1-43-loaded synaptic vesicles during stimulation was computed by fitting the data from 20 s through 140 s to a single exponential decay function using a custom-made MATLAB program. The fitting with R^2^ > 0.5 was used to compare destaining time constants between WT and HD striatal neurons. To perform imaging experiments of FM 4-64 loaded synaptic vesicles in VGLUT1-mCherry-expressing neurons in striatal culture, we transfected striatal neurons with a construct encoding VGLUT1-mCherry (a gift from Dr. Seok-Kyu Kwon at Korea Institute of Science and Technology (KIST)) at DIV9 using Lipofectamine 2000 (11668019, Thermo Fisher Scientific) as previously described (Dalby et al., 2004). Real-time imaging experiments and analyses of FM 4-64-loaded synaptic vesicles in VGLUT1-mCherry-expressing neurons in striatal culture were performed as described in the above.

### Imaging of CypHer5E-labeled inhibitory synaptic vesicles

The imaging experiments of CypHer5E-labeled inhibitory synaptic vesicles were performed similarly to FM 1-43-loaded synaptic vesicles. CypHer5E-labeled antibodies against the luminal domain of vesicular GABA transporter (VGAT) (VGAT–CypHer5E) were loaded into inhibitory synaptic vesicles in cultured striatal neurons by applying 1,200 field stimuli at 10 Hz for 120 s in the presence of 13 nM VGAT–CypHer5E (131 103CpH (Synaptic System)) in a sample chamber at 37°C using a platinum electrode wired to a stimulator. The sample chamber was perfused for 10 min with ACSF solution. Fluorescence images of VGAT–CypHer5E-labeled synaptic vesicles were obtained with ZT640rdc-UF1 (Chroma Technology) and an ET690/50M (Chroma Technology). A 640-nm laser (OBIS 640 (Coherent Inc.)) was used to excite VGAT–CypHer5E. The stimulator was synchronized through Axon Digidata 1550 (Molecular Devices) and the EMCCD camera to trigger field stimulation at 10 Hz for 120 s. The analyses of CypHer5E-labeled synaptic vesicles were performed similarly as those of FM1-43-loaded synaptic vesicles. The fitting with R^2^ > 0.5 was used to compare exocytosis time constants of CypHer5E-labeled synaptic vesicles between WT and HD striatal neurons.

### Analysis

The fluorescence intensities of FM 1-43-loaded and FM 4-64-loaded synaptic vesicles and VGAT-CypHer5E-labeled inhibitory synaptic vesicles within ROIs were measured with MetaMorph. Numerical data are presented as mean ± *standard error of the mean* (SEM). The independent two-tailed Student’s *t*-test and Mann–Whitney U test were used to determine statistical differences between HD and WT neurons. A *p-*value lower than 0.05 (*p* < 0.05) was considered as significant.

## Results

### Medium spiny neurons (MSNs) were predominant in cultured striatal neurons

It was suggested that neurodegeneration in the striatum of HD is associated with abnormal synaptic transmission in striatal neurons (Milnerwood and Raymond, 2010; Raymond, 2016; Tyebji and Hannan, 2017; Barry et al., 2022; Cepeda and Levine, 2022). However, synaptic transmission in HD striatal neurons remains elusive. Particularly, exocytosis of synaptic vesicles at single presynaptic terminals in HD striatal neurons is poorly understood yet. To investigate whether exocytosis of synaptic vesicles is impaired at single presynaptic terminals in HD striatal neurons, we first cultured striatal neurons from zQ175 mice, which contain more than 180 CAG repeats and show the late onset and slow progression of HD symptoms (Heikkinen et al., 2012; Menalled et al., 2012).

**FIGURE 1:**
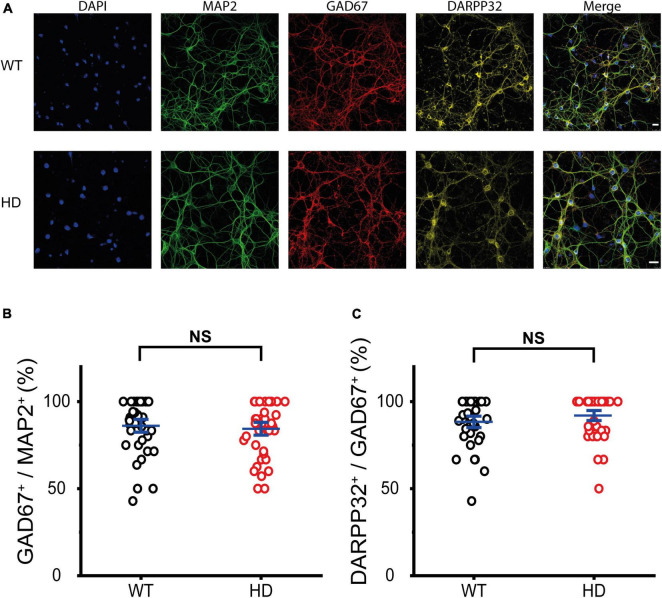
Cultured striatal neurons were predominantly medium spiny neurons (MSNs). **(A)** Representative confocal images of WT and HD cultured striatal neurons immunostained for MAP2 (green), GAD67 (red), and DARPP32 (yellow). Nuclei were counterstained with DAPI (blue). WT and HD cultured striatal neurons were isolated from WT and heterozygous zQ175 mice and were grown on coverslips, respectively. The scale bars represent 20 μm. **(B)** Percentage of GAD67-positive cells in MAP2-positive cells, which represents the ratio of the inhibitory neurons in cultured striatal neurons. The percentage was not significantly different between WT and HD cultured striatal neurons (*p* = 0.62, independent two-tailed Student’s *t*-test). **(C)** Percentage of DARPP32-positive cells in GAD67-positive cells, which represents the ratio of the MSNs. A high percentage of DARPP32-positive cells implies that cultured striatal neurons were predominantly MSNs. The percentage showed no significant difference between WT and HD striatal neurons (*p* = 0.21, independent two-tailed Student’s *t*-test).

First, we examined the identity of cultured WT and HD striatal neurons, which were isolated from striatal tissue of *postnatal* day 0 (P0) WT and heterozygous mice respectively and then were grown until mature synapses were formed. Mature striatal neurons were fixed and then immunostained with antibodies against microtubule-associated protein 2 (MAP2, a marker for neurons), glutamic acid decarboxylase 67 (GAD67, a marker for inhibitory neurons) and dopamine- and adenosine-3′,5′-monophosphate (cAMP)-regulated phosphoprotein of molecular weight 32 kDa (DARPP-32, a marker for MSNs). Representative confocal images of immunostained WT and HD striatal neurons co-stained with DAPI are shown in Figure 1A. Colocalization analyses between MAP2 and GAD67 immunoreactivity revealed that cultured striatal neurons were predominately inhibitory (i.e., GAD67-positive) with no statistical difference between WT and HD neurons (86 ± 2.5% (*n* = 39 images) versus 84 ± 2.5% (*n* = 40), *p* = 0.62 from independent two-tailed Student’s *t*-test) (Figure 1B). To further examine the identity of these cultured striatal neurons, we analyzed immunoreactivity between GAD67 and DARPP-32. The analyses showed that inhibitory neurons in our cultured striatal neurons were predominantly MSNs with no statistical difference between WT and HD neurons (88 ± 2.2% (*n* = 39) versus 92 ± 2.0% (*n* = 40), *p* = 0.21 from independent two-tailed Student’s *t*-test) (Figure 1C). We also measured the percentage of excitatory neurons in our cultured striatal neurons using antibodies against vesicular glutamate transporter 1 (VGLUT1, a marker for excitatory neurons) and found that excitatory neurons were less than 10% of the neurons in the striatal cultures (9.5 ± 0.44% (*n* = 38) versus 8.3 ± 0.47% (*n* = 46), *p* = 0.058 from independent two-tailed Student’s *t*-test) (Supplementary Figure 1). Thus, immunostaining results confirmed that our cultured striatal neurons were predominantly MSNs, which are known to be the principal neurons in the striatum (Kreitzer, 2009) and to undergo neurodegeneration in HD (Vonsattel and DiFiglia, 1998). Thus, our cultured striatal neurons were suitable for investigating the mechanisms of selective neurodegeneration in the striatum of HD.

### Exocytosis of synaptic vesicles was altered at single presynaptic terminals of HD striatal neurons

Then, we examined whether exocytosis of synaptic vesicles is altered at presynaptic terminals of HD striatal neurons. Synaptic vesicles at presynaptic terminals of striatal neurons were loaded with FM 1-43 (a lipophilic styryl dye) using a train of 1,200 electrical field stimuli at 10 Hz for 120 s, which is widely used to label the total recycling pool (TRP) of synaptic vesicles (Ryan and Smith, 1995; Zhang et al., 2007; Welzel et al., 2011; Marra et al., 2012; Park et al., 2012, 2021; Chen et al., 2018; Qin et al., 2019; Chen S. et al., 2021). After loading, we perfused ACSF solution to remove extracellular and plasma membrane-bound FM 1-43.

**FIGURE 2:**
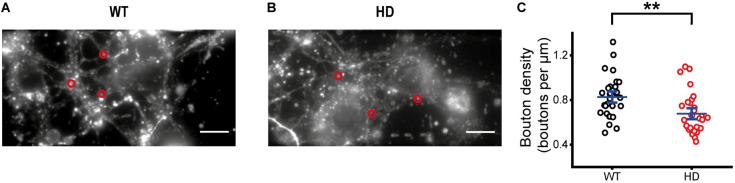
Decreased bouton density in HD striatal neurons. **(A)** Representative image of synaptic vesicles loaded with FM 1-43 in WT striatal neurons. Red circles indicate ROIs representing presynaptic terminals, which were analyzed for exocytosis of FM 1-43. The scale bar represents 10 μm. **(B)** Representative image of synaptic vesicles loaded with FM 1-43 in HD striatal neurons. **(C)** Bouton density in WT (*N* = 6 experiments) and HD (*N* = 8) striatal neurons. **p < 0.01 (Mann–Whitney U test).

Representative fluorescence images of FM 1-43-loaded synaptic vesicles at presynaptic terminals of WT and HD cultured striatal neurons after extensive washing are shown in Figures 2A, B. Fluorescence intensities of FM 1-43-loaded synaptic vesicles were measured within a fixed ROI (Harata et al., 2001; Jordan et al., 2005; Chen et al., 2018), which encircles a single isolated bright spot called a bouton. Single boutons are likely to represent single presynaptic terminals containing FM 1-43-loaded synaptic vesicles. Bouton density was estimated by counting boutons containing FM 1-43-loaded synaptic vesicles along axons. Bouton density in HD striatal neurons was significantly smaller than that in WT neurons (0.68 ± 0.033 boutons/μm for HD neurons (*N* = 8 experiments) versus 0.83 ± 0.034 boutons/μm for WT neurons (*N* = 6), *p* = 0.0012 from Mann–Whitney U test)) (Figure 2C), suggesting that the mutant huntingtin protein decreases the number of functional presynaptic terminals in HD striatal neurons.

**FIGURE 3:**
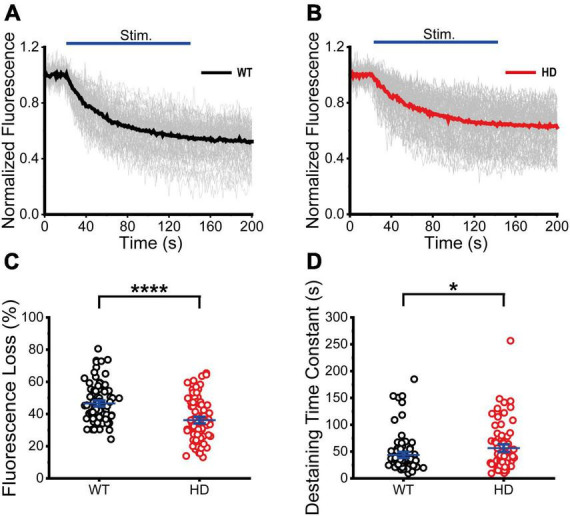
Exocytosis of synaptic vesicles in HD striatal neurons was altered. **(A)** Time courses of normalized fluorescence intensity of FM 1–43-loaded synaptic vesicles during 1,200 electrical stimuli in WT striatal neurons (*n* = 90 boutons, *N* = 12 experiments). The thicker line represents the average normalized fluorescence. The blue line represents 1,200 electrical field stimuli applied at 10 Hz for 120 s. **(B)** Time courses of normalized fluorescence intensity of FM 1–43-loaded synaptic vesicles during 1,200 electrical stimuli in HD striatal neurons (*n* = 87, *N* = 15). **(C)** Percentage of fluorescence loss of FM 1–43-loaded synaptic vesicles in WT and HD striatal neurons. **(D)** Destaining time constant of FM 1–43-loaded synaptic vesicles in WT and HD striatal neurons. *p < 0.05 and ****p < 0.0001 (Mann–Whitney U test).

Next, we performed real-time imaging experiments of FM 1-43-loaded synaptic vesicles at single presynaptic terminals during 1,200 electrical stimuli to measure synaptic vesicle exocytosis. We analyzed fluorescence intensities of isolated boutons that showed destaining upon electrical field stimulation in order to exclude boutons that were labeled by spontaneous activity or non-specific binding (Chen et al., 2018). The decreased fluorescence intensity of FM 1-43 in each ROI after stimulation reflects exocytosis of FM 1-43-loaded synaptic vesicles at single presynaptic terminals. The normalized fluorescence intensity was calculated as the ratio of the fluorescence intensity of FM 1-43 in each ROI with respect to the baseline fluorescence intensity (the average intensity before stimulation) of FM 1-43 (Chen et al., 2018). Figures 3A, B shows time courses of normalized fluorescence intensity at single presynaptic terminals in WT (Figure 3A) and HD (Figure 3B) striatal neurons during 1,200 electrical stimuli. As shown in Figures 3A, B, 1,200 electrical stimuli induced rapid exocytosis of FM 1-43-loaded synaptic vesicles at single presynaptic terminals in both WT and HD striatal neurons. Individual time courses showed heterogeneous exocytosis at single presynaptic terminals. The amount of fluorescence loss at single presynaptic terminals in striatal neurons was highly varied and the coefficient of variation (CV) was 25% and 36% in WT and HD striatal neurons, respectively. However, fluorescence loss after 1,200 electrical stimuli was significantly lower in HD striatal neurons compared to WT striatal neurons (36 ± 1.4% (*n* = 87 boutons, *N* = 15 experiments for HD) versus 47 ± 1.2% (*n* = 90 boutons, *N* = 12 for WT), *p* = 1.4E-7 from Mann–Whitney U test) (Figure 3C). Considering that release probability is in proportion to the relative amount of exocytosed FM dyes (Murthy et al., 1997; Branco et al., 2008; Branco and Staras, 2009; Daniel et al., 2009), decreased fluorescence loss at single presynaptic terminals of HD striatal neurons suggests that the mutant huntingtin protein reduces the release probability of synaptic vesicles at single presynaptic terminals of HD striatal neurons. Furthermore, we measured the kinetics of synaptic vesicle exocytosis by fitting the time courses of fluorescence loss to a single exponential decay function (Richards et al., 2005; Daniel et al., 2009; Chen et al., 2018). Figure 3D shows that the destaining time constant of FM 1-43 in HD was larger compared with WT neurons (56 ± 4.8 s (*n* = 80 boutons) for HD versus 44 ± 3.6 s (*n* = 87 boutons) for WT, *p* = 0.032 from Mann–Whitney U test), implying slower destaining rate of synaptic vesicles at single presynaptic terminals of HD striatal neurons. Taken together, our results suggest that the mutant huntingtin protein decreases the number of functional presynaptic terminals and exocytosis at single presynaptic terminals of cultured striatal neurons of HD.

### Exocytosis of inhibitory synaptic vesicles was altered in HD striatal neurons

**FIGURE 4:**
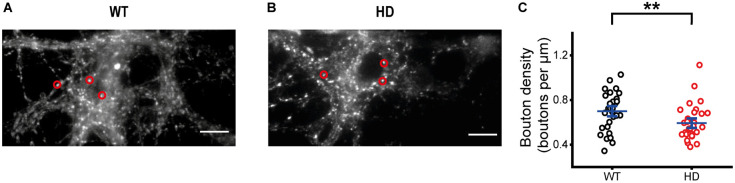
HD striatal neurons exhibited reduced inhibitory bouton density. **(A)** Representative image of inhibitory synaptic vesicles labeled with VGAT-CypHer5E in WT striatal neurons. Red circles indicate ROIs representing inhibitory presynaptic terminals, which were analyzed for exocytosis of VGAT-CypHer5E. The scale bar represents 10 μm. **(B)** Representative image of inhibitory synaptic vesicles labeled with VGAT-CypHer5E in HD striatal neurons. **(C)** Inhibitory bouton density in WT (*N* = 6 experiments) and HD (*N* = 8) striatal neurons. **p < 0.01 (Mann–Whitney U test).

Since our immunostaining results showed that the majority of our cultured striatal neurons were inhibitory neurons and exocytosis of synaptic vesicles at single presynaptic terminals of HD striatal neurons was altered, we further examined whether exocytosis of inhibitory synaptic vesicles is altered at single presynaptic terminals of HD striatal neurons. To measure exocytosis of inhibitory synaptic vesicles at single presynaptic terminals, we specifically labeled inhibitory synaptic vesicles with CypHer5E-labeled antibodies against the luminal domain of vesicular GABA transporter (VGAT–CypHer5E) (Chen et al., 2018; Park et al., 2021) because CypHer5E shows maximum fluorescence at acidic pHs and drastic fluorescence decrease at neutral pHs (Beletskii et al., 2005; Hua et al., 2011). Similar to FM 1-43 staining, we stimulated neurons with 1,200 electrical stimuli to label inhibitory synaptic vesicles from the TRP with VGAT–CypHer5E. Figures 4A, B show representative fluorescence images of VGAT–CypHer5E-labeled inhibitory synaptic vesicles at presynaptic terminals of WT (Figure 4A) and HD striatal neurons (Figure 4B). Inhibitory bouton density measured by VGAT–CypHer5E-labeled synaptic vesicles in HD striatal neurons was smaller than that in WT neurons (0.59 ± 0.028 boutons/μm for HD neurons (*N* = 8 experiments)) versus 0.70 ± 0.030 boutons/μm for WT neurons (*N* = 6), *p* = 0.0063 from Mann–Whitney U test) (Figure 4C), suggesting that the mutant huntingtin protein decreases the number of functional inhibitory presynaptic terminals in HD striatal neurons.

**FIGURE 5:**
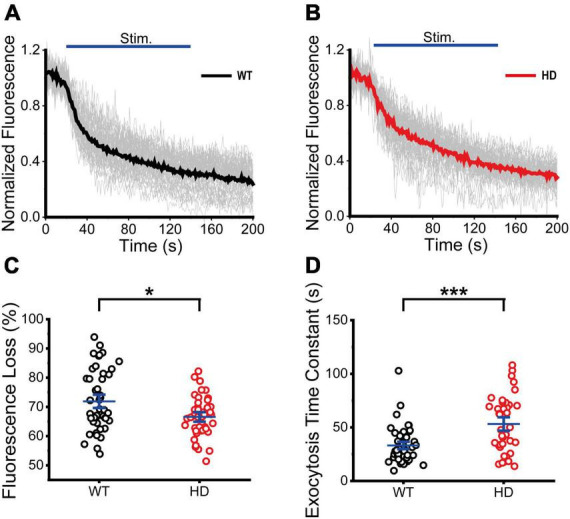
Exocytosis of inhibitory synaptic vesicles in HD striatal neurons was altered. **(A)** Time courses of normalized fluorescence intensity of VGAT-CypHer5E-labeled inhibitory synaptic vesicles during 1,200 electrical field stimuli in WT striatal neurons (*n* = 47 boutons, *N* = 5 experiments). The thicker line represents average normalized fluorescence. The blue line represents 1,200 electrical field stimuli applied at 10 Hz for 120 s. **(B)** Time courses of normalized fluorescence intensity of VGAT-CypHer5E-labeled inhibitory synaptic vesicles during 1,200 electrical stimuli in HD striatal neurons (*n* = 41, *N* = 5). **(C)** Percentage of fluorescence loss of VGAT-CypHer5E-labeled inhibitory synaptic vesicles in WT and HD striatal neurons. **(D)** Exocytosis time constant of VGAT-CypHer5E-labeled inhibitory synaptic vesicles in WT and HD striatal neurons. *p < 0.05 and ***p < 0.001 (Mann–Whitney U test).

Next, we performed real-time imaging experiments of VGAT–CypHer5E-labeled inhibitory synaptic vesicles at single presynaptic terminals during 1,200 electrical stimuli to measure exocytosis of inhibitory synaptic vesicles. Fluorescence intensities of boutons containing VGAT–CypHer5E-labeled inhibitory synaptic vesicles drastically decreased upon electrical stimulation, reflecting exocytosis of VGAT–CypHer5E-labeled inhibitory synaptic vesicles at single presynaptic terminals. The normalized fluorescence intensity of VGAT–CypHer5E-labeled inhibitory synaptic vesicles was calculated using the same method as FM 1-43. Figures 5A, B show time courses of normalized fluorescence intensity of VGAT–CypHer5E-lableled inhibitory synaptic vesicles at single presynaptic terminals in WT (Figure 5A) and HD (Figure 5B) striatal neurons in a train of 1,200 electrical stimuli. 1,200 electrical stimuli induced rapid exocytosis of VGAT–CypHer5E-labeled inhibitory synaptic vesicles at single presynaptic terminals in both WT and HD striatal neurons. Individual time courses of normalized fluorescence intensity showed heterogeneous exocytosis of VGAT–CypHer5E-labeled inhibitory synaptic vesicles at single presynaptic terminals with CV values of 14% and 10% for WT and HD striatal neurons, respectively. Fluorescence loss after 1,200 electrical stimuli was significantly lower in HD striatal neurons compared to WT striatal neurons (67 ± 1.1% (*n* = 41 boutons, *N* = 5 experiments for HD) versus 72 ± 1.5% (*n* = 47 boutons, *N* = 5 for WT), *p* = 0.040 from Mann–Whitney U test) (Figure 5C). Considering the relationship between release probability and the relative amount of exocytosed VGAT–CypHer5E, decreased fluorescence loss at presynaptic terminals of HD striatal neurons suggests that the mutant huntingtin protein decreases the release probability of inhibitory synaptic vesicles at single presynaptic terminals of HD striatal neurons. Furthermore, we measured the exocytosis rates of inhibitory synaptic vesicles by fitting the time courses of fluorescence loss to a single exponential decay function. Figure 5D shows that the exocytosis time constant of VGAT–CypHer5E-labeled synaptic vesicles in HD striatal neurons was significantly larger than WT neurons (54 ± 4.2 s (*n* = 38 boutons) for HD versus 34 ± 2.4 s (*n* = 49 boutons) for WT, *p* = 2.6E-4 from Mann–Whitney U test), indicating slower exocytosis rate of inhibitory synaptic vesicles at single presynaptic terminals of HD striatal neurons. Given decreased density of functional inhibitory synapses and exocytosis at single presynaptic terminals of striatal neurons expressing the mutant huntingtin protein, we conclude that the mutant huntingtin protein alters inhibitory neurotransmission at single presynaptic terminals of HD striatal neurons. We also examined whether excitatory neurotransmission is altered in excitatory synapses of cultured striatal neurons by performing real-time imaging experiments of FM 4-64-loaded synaptic vesicles in VGLUT1-mCherry-expressing neurons in striatal cultures. We found no significant difference in bouton density, fluorescence loss and destaining time constant between WT and HD striatal neurons expressing VGLUT1-mCherry (Supplementary Figure 2), suggesting that exocytosis of excitatory synaptic vesicles may not be altered in HD striatal neurons expressing VGLUT1-mCherry.

## Discussion

Striatal neurons are the most vulnerable neurons in HD and undergo neurodegeneration in the disease progression of HD (Vonsattel and DiFiglia, 1998). However, the mechanisms of selective neurodegeneration of striatal neurons in HD remain elusive. Here, we used real-time imaging of FM 1-43-loaded synaptic vesicles during electrical field stimulation and showed a decrease in bouton density and exocytosis at single presynaptic terminals of cultured striatal neurons in zQ175 (a knock-in mouse model of HD) mice. Real-time imaging of VGAT-CypHer5E-labeled inhibitory synaptic vesicles revealed reduced inhibitory bouton density and exocytosis of inhibitory synaptic vesicles at single presynaptic terminals of striatal neurons from zQ175 mice. Our results suggest that the mutant huntingtin protein decreases functional inhibitory presynaptic terminals and exocytosis of inhibitory synaptic vesicles at single presynaptic terminals of striatal neurons, leading to impaired inhibitory synaptic transmission in the striatum of HD.

Our findings about the decrease in exocytosis of inhibitory synaptic vesicles at single presynaptic terminals in cultured striatal neurons of zQ175 mice are consistent with recent whole-cell patch clamp results showing decreased current in inhibitory evoked synaptic responses with the decreased readily releasable pool (RRP) in autaptic striatal neurons infected with lentiviruses expressing human huntingtin exon 1 with expanded CAG repeats (97Q-Htt) (Paraskevopoulou et al., 2021). Moreover, our results fit with other papers reporting decreased concentration of GABA in the striatum in several HD mouse models and HD patients (Perry et al., 1973; Spokes et al., 1980; Wójtowicz et al., 2013; Serranilla and Woodin, 2021). Recently, real-time imaging of Synaptophysin-pHluorin showed altered endocytosis of synaptic vesicles during high frequency (40Hz) stimulation in cultured striatal neurons from another knock-in HD mouse model (Q140 mice) (McAdam et al., 2020). These altered exocytosis and endocytosis of synaptic vesicles in striatal neurons of HD might disrupt synaptic transmission in the striatum of HD (Cepeda and Levine, 2022) because exocytosis and endocytosis of synaptic vesicles mediates synaptic transmission (Mochida, 2022; Park et al., 2022). Disrupted synaptic transmission may weaken synaptic connection, which was observed as decreased bouton density in our cultured HD striatal neurons and as decreased spine density in the MSNs in the striatum of symptomatic R6/2 (Cepeda et al., 2013) and zQ175 mice (Indersmitten et al., 2015). Weakened synaptic connection in the striatal circuit could cause abnormal movements in HD patients.

Neurons in the striatum receive excitatory inputs from the cortex and the thalamus through corticostriatal and thalamostriatal synapses (Ding et al., 2008; Hunnicutt et al., 2016; Reiner and Deng, 2018). Destaining experiments of FM dyes demonstrated an increase in exocytosis of synaptic vesicles in cultured cortical neurons (Chen et al., 2018) and brain slices of young HD mice (Joshi et al., 2009). Electrophysiology measurements suggested increased release probability of synaptic vesicles from presynaptic terminals of HD thalamic neurons (Kolodziejczyk and Raymond, 2016). Increased release of excitatory neurotransmitters through corticostriatal and thalamostriatal synapses can play a detrimental role in HD striatal neurons. Moreover, real-time imaging of BDNF-pHluorin revealed deceased release of BDNF in cultured cortical neurons of HD mice (Yu et al., 2018). These increased release of excitatory neurotransmitters from cortical and thalamic neurons, decreased release of BDNF from cortical neurons, and decreased release of inhibitory neurotransmitters in striatal neurons may contribute to neurodegeneration in the striatum of HD.

Mechanisms underlying these decreased functional presynaptic density and exocytosis of inhibitory synaptic vesicles at single presynaptic terminals of striatal neurons expressing the mutant huntingtin protein remain to be elucidated. Recent RNA sequencing of striatal neurons containing the mutant huntingtin protein revealed differentially expressed genes (Paraskevopoulou et al., 2021; Matsushima et al., 2023), which provides possible clues to molecular mechanisms for altered synaptic transmission in HD. Furthermore, the application of single synaptic vesicle tracking methods (Park et al., 2012, 2022; Joensuu et al., 2016; Yu et al., 2016; Chen X. et al., 2021) could help to elucidate whether altered exocytosis of synaptic vesicles is caused by the dynamics of single inhibitory synaptic vesicles in HD striatal neurons.

In summary, we report decreased functional inhibitory presynaptic density and exocytosis of inhibitory synaptic vesicles at single presynaptic terminals during electrical field stimulation in cultured striatal neurons obtained from zQ175 HD mice. Our findings suggest that the mutant huntingtin protein decreases exocytosis of inhibitory synaptic vesicles at single presynaptic terminals of striatal neurons, impairing inhibitory synaptic transmission, eventually leading to neurodegeneration in the striatum of HD. Thus, our work provide a new insight into selective neurodegeneration of vulnerable neurons in HD.

## Data availability statement

The raw data supporting the conclusions of this article will be made available by the authors, without undue reservation.

## Ethics statement

The animal study was reviewed and approved by Hong Kong University of Science and Technology.

## Author contributions

CX, SC, XC, and HP designed the experiments and wrote the manuscript. CX, SC, XC, KH, and HY performed the experiments. CX, SC, XC, KH, and CP analyzed the data. SC provided the analysis programs. CX, SC, XC, S-HL, and HP interpreted the results. All authors contributed to the article and approved the submitted version.
