# Supplementary material for: Altered exocytosis of inhibitory synaptic vesicles at single presynaptic terminals of cultured striatal neurons in a knock-in mouse model of Huntington’s disease
Source: Front Mol Neurosci. 2023 Aug 17;16:1175522. doi: 10.3389/fnmol.2023.1175522 (PMC10470468; doi:10.3389/fnmol.2023.1175522)
Supplement: Supplementary file 1 [file Presentation_1.pdf]

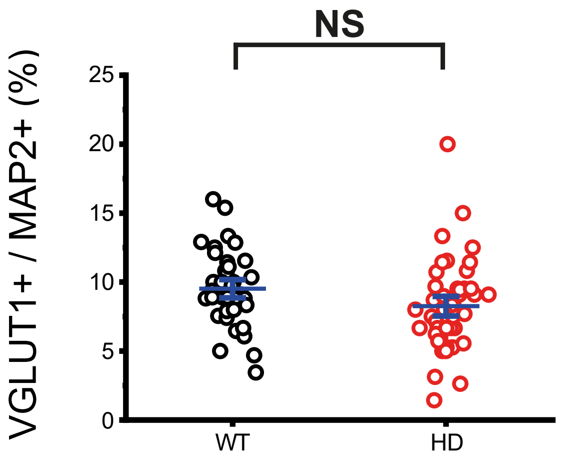

Supplemental Figure S1| Percentage of VGLUT1-positive cells in MAP2-positive cells, which represents the percentage of excitatory neurons in cultured striatal neurons. The percentage of VGLUT1-positive cells was not significantly different between WT (n = 38 images) and HD (n = 46) (p=0.058, independent two-tailed Student's t-test).

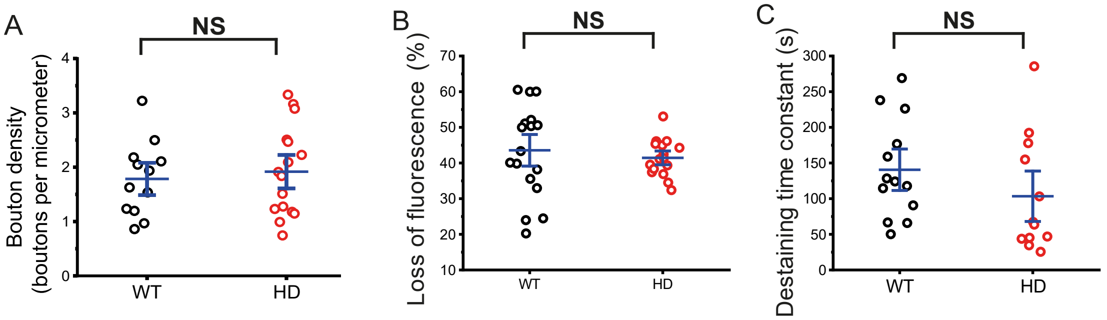

Supplemental Figure S2| Synaptic vesicle exocytosis of VGLUT1-mCherry-expressing striatal neurons loaded with FM 4-64. **(A)** Bouton density loaded with FM 4-64 in WT (N = 3 experiments) and HD (N = 3) striatal neurons expressing VGLUT1-mCherry. Bouton density was not significantly different between WT and HD neurons (*p*=0.80, Mann-Whitney U Test). **(B)** Percentage of fluorescence loss of FM 4-64-loaded synaptic vesicles in WT (n = 18 boutons, N = 3 experiments) and HD (n = 16, N = 3) striatal neurons expressing VGLUT1-mCherry in 1200 external electrical stimuli. The percentage of fluorescence loss was not significantly different between WT and HD neurons (*p*=0.40, Mann-Whitney U Test). **(C)** Destaining time constant of FM 4-64-loaded synaptic vesicles in WT (n = 13, N = 3) and HD (n = 12, N = 3) striatal neurons expressing VGLUT1-mCherry. The destaining time constant of FM 4–64-loaded synaptic vesicles was not significantly different (*p*=0.12, Mann-Whitney U Test).
